# Association between full service and fast food restaurant density, dietary intake and overweight/obesity among adults in Delhi, India

**DOI:** 10.1186/s12889-017-4598-8

**Published:** 2017-07-19

**Authors:** Opal Patel, Safraj Shahulhameed, Roopa Shivashankar, Mohammad Tayyab, Atiqur Rahman, Dorairaj Prabhakaran, Nikhil Tandon, Lindsay M. Jaacks

**Affiliations:** 1 Department of Environmental Health, Rollins School of Public Health, Emory University, Atlanta, GA USA; 2 Public Health Foundation of India and Centre for Control of Chronic Conditions (4Cs), Gurgaon, Haryana India; 3 Department of Geography, Jamia Milia Islamia, New Delhi, India; 4 Department of Endocrinology and Metabolism, All India Institute of Medical Sciences, New Delhi, India; 5 Department of Global Health and Population, Harvard T.H. Chan School of Public Health, Boston, MA USA

**Keywords:** Food outlets, Geographic information system, Body mass index, Obesity, India

## Abstract

**Background:**

The food environment has been implicated as an underlying contributor to the global obesity epidemic. However, few studies have evaluated the relationship between the food environment, dietary intake, and overweight/obesity in low- and middle-income countries (LMICs). The aim of this study was to assess the association of full service and fast food restaurant density with dietary intake and overweight/obesity in Delhi, India.

**Methods:**

Data are from a cross-sectional, population-based study conducted in Delhi. Using multilevel cluster random sampling, 5364 participants were selected from 134 census enumeration blocks (CEBs). Geographic information system data were available for 131 CEBs (*n* = 5264) from a field survey conducted using hand-held global positioning system devices. The number of full service and fast food restaurants within a 1-km buffer of CEBs was recorded by trained staff using ArcGIS software, and participants were assigned to tertiles of full service and fast food restaurant density based on their resident CEB. Height and weight were measured using standardized procedures and overweight/obesity was defined as a BMI ≥25 kg/m^2^.

**Results:**

The most common full service and fast food restaurants were Indian savory restaurants (57.2%) and Indian sweet shops (25.8%). Only 14.1% of full service and fast food restaurants were Western style. After adjustment for age, household income, education, and tobacco and alcohol use, participants in the highest tertile of full service and fast food restaurant density were less likely to consume fruit and more likely to consume refined grains compared to participants in the lowest tertile (both *p* < 0.05). In unadjusted logistic regression models, participants in the highest versus lowest tertile of full service and fast food restaurant density were significantly more likely to be overweight/obese: odds ratio (95% confidence interval), 1.44 (1.24, 1.67). After adjustment for age, household income, and education, the effect was attenuated: 1.08 (0.92, 1.26). Results were consistent with further adjustment for tobacco and alcohol use, moderate physical activity, and owning a bicycle or motorized vehicle.

**Conclusions:**

Most full service and fast food restaurants were Indian, suggesting that the nutrition transition in this megacity may be better characterized by the large number of unhealthy Indian food outlets rather than the Western food outlets. Full service and fast food restaurant density in the residence area of adults in Delhi, India, was associated with poor dietary intake. It was also positively associated with overweight/obesity, but this was largely explained by socioeconomic status. Further research is needed exploring these associations prospectively and in other LMICs.

**Electronic supplementary material:**

The online version of this article (doi:10.1186/s12889-017-4598-8) contains supplementary material, which is available to authorized users.

## Background

Cardiovascular diseases (CVD) account for one-fourth of deaths in India [1] and CVD and diabetes are predicted to cost India $2.32 trillion USD from 2012 to 2030 (including both direct costs of treatment and indirect costs of lost labor supply due to mortality) [2]. Tobacco use, unhealthy diets, sedentary lifestyles, and overweight/obesity are among the leading risk factors for CVD worldwide and are becoming increasingly common in India [3]. Interventions and policies for prevention of these risk factors are urgently needed.

One target for preventive efforts has been the food environment [4, 5], defined as the physical presence of food and food stores that affects people’s dietary intake [6]. In particular, the presence of full service and fast food restaurants (the latter including both self-service and carry-out venues) may lead to weight gain [7, 8] by providing large portions of high-energy foods with low nutrient density [9, 10]. Few studies have quantified the association between the presence of restaurants and dietary intake in low- and middle-income countries (LMICs). One study in Brazil found no association between fast food restaurant density and sugar-sweetened beverage consumption [11]. No studies in LMICs have evaluated restaurant density in association with more comprehensive measures of dietary intake.

Similar to the research on dietary intake, to date, most of the research on the food environment and overweight/obesity has been conducted in the United States [12, 13]. Results of studies conducted in LMICs have been inconclusive. A cross-sectional survey in China found a positive association between number of fast food restaurants and overweight/obesity among adolescents [14]. Another study in China also found a positive association between changes in number of Western fast food restaurants and waist-to-height ratio, but no association with body mass index (BMI) [15]. A study of school children in Taiwan found fast food restaurant density was associated with BMI in boys, but not in girls [16]. In contrast, other studies in China [17], Brazil [11], and Japan [18] found no association between proximity to restaurants and overweight/obesity.

Thus, it is unclear whether the presence of full service and fast food restaurants is associated with dietary intake or overweight/obesity in LMICs. Moreover, no study has evaluated this association in urban India where interventions and policies to address overweight/obesity are urgently needed. Extrapolation from studies conducted in the United States or other high-income countries may lead to incorrect conclusions regarding the effects of the urban food environment. The aim of this study was to evaluate the relationship between full service and fast food restaurants assessed using hand-held global positioning system (GPS) devices and 1) dietary intake and 2) overweight/obesity (BMI ≥25 kg/m^2^) in a representative sample of Delhi, India.

## Methods

### Sample population

Data are from the Centre for Cardiometabolic Risk Reduction in South-Asia (CARRS) study [19]. CARRS collected baseline data in 2010-2011 from three cities in South Asia: Delhi and Chennai in India, and Karachi in Pakistan. CARRS used a multistage probability sampling to select representative samples of the target populations. The response rate was 94.7% for questionnaire completion. This analysis included only participants from Delhi.

The 2001 Census was used to develop the CARRS sampling frame [19]. In 2001, Delhi was divided into nine districts of varying sizes and population, and each district (except New Delhi) was divided into three subdivisions. Subdivisions were further divided into urban and rural areas, and urban areas were further divided into wards and wards into census enumeration blocks (CEBs). The areas covered in CARRS are under the purview of the Municipal Corporation of Delhi. Three districts (New Delhi, North, and South West districts) were excluded from CARRS. New Delhi and North Districts are primarily commercial areas and the South West district comprises defense personnel, marshy agricultural area, and expatriates who were likely to leave the country during the study period. CARRS randomly selected 20 wards and 5-7 CEBs within each ward totaling to 134 CEBs.

### Outcome assessment

Dietary intake was evaluated using a 26-item food propensity questionnaire adapted from the INTERHEART study [20]. Portion size information was not collected, only the frequency of consumption (never or less than once a month, per month, per week, or per day) over the past year, which was standardized to consumption per day and categorized into four categories: never or <1/month, ≥1/month but <1/week, ≥1/week but <1/day, and ≥1/day [21]. A total of 15 food groups were analyzed: meat, poultry, fish and shellfish, eggs, dairy, nuts and seeds, legumes and pulses, fruit, vegetables, whole grains, refined grains, desserts, deep-fried foods, fruit juice, and sugar-sweetened beverages.

Trained staff used standardized procedures to measure weight and height [19]. BMI was calculated as weight (kg) divided by height-squared (m^2^). Obesity including overweight was defined as BMI ≥25 kg/m^2^ [22]. The South Asian cut-point for overweight/obese of BMI ≥23 kg/m^2^ was also evaluated [23]. Missing weight data (33.7%) were imputed using the multiple imputation chained equation approach in Stata v12.0 (StataCorp LP, College Station, TX).

### Food environment assessment

Food environment was defined as consisting of full service and fast food restaurants in a participant’s residential neighborhood. Full service restaurants were those that had sit down service available to customers, and fast food restaurants were those with minimal table service. Participant household locations were geocoded by field surveyors using hand-held GPS devices and the locations verified using Google Earth satellite imagery. The geocoded households were incorporated into a geographic information system (GIS) environment. Participant neighborhood was created by first locating and marking all households in a CEB. A CEB polygon was created linking the outermost households in each CEB while including all households within the CEB. A 1-km buffer was created around the boundaries of each CEB polygon. We used 1-km buffers as this was considered normal walking distance in the community and is also consistent with previous studies conducted in Asia [15, 18]. This 1-km buffer was defined as the unit of analysis for all neighborhood environment characteristics [24].

The location and name of all full service and fast food restaurants within each CEB neighborhood was recorded by the field surveyors. Restaurants were defined as *pucca* structures selling cooked food, e.g., permanent establishments built with conventional construction materials. Street food vendors were not included due to our inability to record the number of street food vendors that exist in an area since this varies hour-to-hour and day-to-day. Outlets were categorized based on their name into one of four categories: Indian sweets (e.g., Aggarwal and Bikaner Sweets), Indian savory (e.g., Apni Rasoi restaurant and Moti Mahal restaurant), Western (e.g., KFC, Subway, Pizza Hut, and McDonald’s), and Chinese. In sensitivity analyses, when the association of each of these categories with overweight/obesity was evaluated separately, results were consistent across the categories, thus, the exposure variable in models included all full service and fast food restaurants combined.

To account for variation in CEB size (mean [SD] of 3.5 km^2^ [0.16 km^2^]), the number of full service and fast food restaurants in each CEB was divided by the size of the CEB in km-squared, thus the exposure variable included in the models was full service and fast food restaurant density (number of outlets per km^2^). Full service and fast food restaurant density was specified as tertiles in the models to account for the non-normal distribution. In order to visualize the association between full service and fast food restaurants and BMI, we overlaid a heat map of BMI levels with the GPS coordinates of the full service and fast food restaurants. All GIS analyses were carried out using ESRI ArcGIS software version 10.1, year of release 2012 (Environmental Systems Research Institute, Redlands, CA). All GIS data were collected in 2013-2014.

### Covariate assessment

Sociodemographic information was collected by trained interviewers using questionnaires, and included age, sex, marital status (unmarried or married), educational status (up to primary school; high school up to secondary school; or graduate level or higher), employment status (not working, which includes students, housewives, retired; unskilled/semi-skilled; trained/skilled; or white collar), and household income (<10,000 INR; 10,000-20,000 INR; or >20,000 INR). Additional sociodemographic and lifestyle behavior variables included car ownership (yes or no), motorized bike ownership (yes or no), bicycle ownership (yes or no), vegetarian diet (yes or no), and level of moderate physical activity (none; <150 min/week; or ≥150 min/week).

### Statistical analysis

All analyses were conducted in SAS software version 9.4 (SAS Institute Inc., Cary, NC). Analysis of variance (continuous variables) and Chi-square tests (binary and categorical variables) were used to evaluate the association between full service and fast food restaurant density and sociodemographic characteristics.

The association between full service and fast food restaurant density and dietary intake was evaluated using multinomial logistic regression. Two models were estimated for each dietary intake variable (e.g., 15 food groups): model 1 was unadjusted and model 2 was adjusted for age, household income, education, and tobacco and alcohol use.

The association between full service and fast food restaurant density and overweight/obesity was estimated using logistic regression. Linear regression was used to estimate the association between full service and fast food restaurant density and BMI. SAS PROC MIANALYZE was used to calculate the average of the 10 complete-data estimates from the multiple imputations. Consistent with standard definitions of a confounder [25], sociodemographic characteristics that were significantly associated with full service and fast food restaurant density, and also risk factors for overweight/obesity, were included in adjusted models. Four models were estimated: model 1 was unadjusted; model 2 was adjusted for age, household income, and education; model 3 was adjusted for the variables in model 2 plus tobacco and alcohol use; and model 4 was adjusted for the variables in model 3 plus level of moderate physical activity and ownership of a bicycle, car, or motorized bike. Because dietary intake was thought to be a mediator of the association between full service and fast food restaurant density and overweight/obesity, we did not include it in the models [26].

Finally, as an exploratory analysis to improve interpretation of results, we used a Chi-square test to evaluate the association between full service and fast food restaurant density and median household wealth index of CEBs. Briefly, the household wealth index was derived using principal component analysis based on household amenities (separate cooking room and toilet facilities) and assets (television, refrigerator, washing machine, microwave, mixer-grinder, mobile phone, DVD player, computer, car, motorized bike, and bicycle) [27]. The median household wealth index was then calculated for each CEB and then CEBs were categorized into tertiles based on these values. In addition to this Chi-square test, we present descriptive statistics for the mean (SD) BMI of participants in each cell of the 3 × 3 table of CEB full service and fast food restaurant density tertile by CEB median household wealth index tertile.

## Results

A total of 5364 adults in 134 CEBs participated in CARRS at the Delhi site. Three CEBs did not have GIS data, and therefore participants from those CEBs (*n* = 100) were not included in this analysis. The final sample size was 5264. The number of full service and fast food restaurants in the CEBs ranged from 1 to 46 with a mean (SD) of 11 (10) outlets. Additional file 1 provides the distribution of BMI and full service and fast food restaurants in urban Delhi. The most common full service and fast food restaurants were Indian sweet shops and Indian savory restaurants: 25.8% Indian sweets (e.g., Aggarwal and Bikaner Sweets, etc.), 57.2% Indian savory (e.g., Apni Rasoi restaurant and Moti Mahal restaurant, etc.), 14.1% Western (e.g., KFC, Subway, Pizza Hut, McDonald’s, etc.), and 2.9% Chinese. The mean (SD) full service and fast food restaurant density for tertile 1 was 0.97 (0.53) outlets per km^2^; for tertile 2 was 2.24 (0.38) outlets per km^2^; and for tertile 3 was 6.18 (2.78) outlets per km^2^.

All sociodemographic characteristics, except sex, were significantly associated with full service and fast food restaurant density (Table 1). Participants in the highest tertile of full service and fast food restaurant density were older and more likely to have a household income >20,000 INR, a white collar employment, graduate level or higher education, never used tobacco, and own a motorized bike or car compared to participants in the lowest tertile.

**Table 1:** Sociodemographic characteristics of participants according to full service and fast food restaurant density (*N* = 5264)

	Full service and fast food restaurant density (tertiles)			
	1 (*n* = 1737)	2 (*n* = 1782)	3 (*n* = 1745)	*P*-value^a^
Age (years)	43.7 (12.8)	44.1 (13.7)	45.6 (13.8)	<0.0001
Sex				0.97
Male	50.0 (869)	50.2 (894)	49.7 (868)	
Female	50.0 (868)	49.8 (888)	50.3 (877)	
Marital status				0.009
Unmarried	10.1 (175)	10.4 (186)	13.1 (228)	
Married	89.9 (1562)	89.6 (1596)	86.9 (1517)	
Household income				<0.0001
< 10,000 INR	57.3 (989)	56.9 (1008)	32.7 (566)	
10,000-20,000 INR	20.5 (354)	20.4 (362)	26.5 (459)	
> 20,000 INR	22.1 (382)	22.7 (402)	40.8 (706)	
Employment status				<0.0001
Not working	51.2 (889)	52.1 (928)	50.8 (887)	
Unskilled/semi-skilled	19.0 (330)	20.6 (367)	14.7 (257)	
Skilled/trained	24.8 (430)	24.2 (432)	25.6 (447)	
White collar	5.1 (88)	3.1 (55)	8.8 (154)	
Education				<0.0001
Up to Primary School	24.5 (425)	25.9 (461)	13.7 (239)	
High School up to Secondary School	53.3 (926)	54.0 (962)	53.1 (926)	
Graduate Level or Higher	22.2 (386)	20.2 (359)	33.2 (580)	
Tobacco use				0.0003
Never used	71.9 (1249)	73.5 (1310)	77.6 (1354)	
Used in past (but not currently)	1.8 (32)	2.2 (39)	2.5 (43)	
Currently using	26.3 (456)	24.3 (433)	19.9 (348)	
Alcohol use				0.0006
Never used	82.7 (1437)	85.1 (1517)	79.5 (1388)	
Used in past or currently use occasionally	12.8 (222)	11.3 (201)	15.0 (261)	
Currently using	4.5. (78)	3.6 (64)	5.5 (96)	
Motorized bike				<0.0001
Yes	46.0 (799)	46.2 (822)	58.9 (1027)	
No	54.0 (937)	53.9 (959)	41.2 (718)	
Car				<0.0001
Yes	24.4 (423)	20.7 (368)	43.8 (765)	
No	75.6 (1313)	79.3 (1413)	56.2 (980)	
Bicycle				0.0006
Yes	30.2 (524)	24.5 (437)	26.3 (458)	
No	69.8 (1212)	75.5 (1344)	73.8 (1287)	
Vegetarian				<0.0001
Yes	54.5 (947)	57.7 (1029)	48.4 (845)	
No	45.5 (790)	42.3 (753)	51.6 (900)	
Moderate physical activity				0.006
None	55.3 (959)	59.9 (1065)	59.4 (1034)	
< 150 min/week	22.7 (393)	18.0 (320)	19.1 (332)	
≥ 150 min/week	22.1 (383)	22.2 (394)	21.5 (375)	

Values are mean (SD) or percent (n)

^a^ *P*-value from analysis of variance (continuous variables) or Chi-square test (binary and categorical variables) of association between full service and fast food restaurant density and sociodemographic characteristics

After adjustment for confounders (age, household income, education, and tobacco and alcohol use), participants in the highest tertile of full service and fast food restaurant density were less likely to consume meat, fish and shellfish, eggs, and fruit, and more likely to consume refined grains compared to participants in the lowest tertile (Table 2). Participants in the highest tertile of full service and fast food restaurant density were more likely to consume desserts, fruit juice, and sugar-sweetened beverages (all *p* < 0.05) in unadjusted models, but these effects we attenuated after adjustment for confounders. Participants in the middle tertile of full service and fast food restaurant density, after adjustment for confounders, were more likely to consume meat, poultry, eggs, refined grains, and desserts, and less likely to consume fruit juice and sugar-sweetened beverages compared to participants in the lowest tertile. No significant associations were observed for dairy, nuts and seeds, legumes and pulses, whole grains, or deep-fried foods after adjustment for confounders.

**Table 2:** Association between full service and fast food restaurant density (number per km^2^) and dietary intake among adults living in Delhi, India (*N* = 5264)

	Full service and fast food restaurant density (tertiles)		
	1 (*n* = 1737)	2 (*n* = 1782)	3 (*n* = 1745)
Meat			
Never or <1/month	Referent	Referent	Referent
≥ 1/month but <1/week	Referent	1.22 (1.02, 1.46)	1.16 (0.96, 1.40)
≥ 1/week but <1/day	Referent	1.18 (1.00, 1.40)	0.79 (0.66, 0.95)
≥ 1/day	Referent	1.56 (0.86, 2.80)	0.63 (0.30, 1.34)
Poultry			
Never or <1/month	Referent	Referent	Referent
≥ 1/month but <1/week	Referent	1.23 (1.03, 1.46)	1.04 (0.87, 1.25)
≥ 1/week but <1/day	Referent	1.07 (0.91, 1.27)	0.90 (0.75, 1.07)
≥ 1/day	Referent	2.57 (0.99, 6.71)	2.12 (0.79, 5.72)
Fish and shellfish			
Never or <1/month	Referent	Referent	Referent
≥ 1/month but <1/week	Referent	0.95 (0.80, 1.13)	0.79 (0.65, 0.95)
≥ 1/week but <1/day	Referent	1.02 (0.81, 1.27)	0.72 (0.56, 0.92)
≥ 1/day	Referent	1.95 (0.78, 4.87)	1.00 (0.36, 2.78)
Eggs			
Never or <1/month	Referent	Referent	Referent
≥ 1/month but <1/week	Referent	1.24 (1.01, 1.51)	1.19 (0.97, 1.46)
≥ 1/week but <1/day	Referent	1.10 (0.94, 1.29)	1.01 (0.86, 1.20)
≥ 1/day	Referent	1.14 (0.84, 1.55)	0.69 (0.49, 0.97)
Dairy			
Never or <1/month	Referent	Referent	Referent
≥ 1/month but <1/week	Referent	0.94 (0.73, 1.21)	1.15 (0.88, 1.50)
≥ 1/week but <1/day	Referent	0.97 (0.80, 1.16)	1.19 (0.98, 1.45)
≥ 1/day	Referent	1.05 (0.88, 1.24)	1.09 (0.91, 1.30)
Nuts and seeds			
Never or <1/month	Referent	Referent	Referent
≥ 1/month but <1/week	Referent	1.06 (0.89, 1.27)	0.97 (0.80, 1.17)
≥ 1/week but <1/day	Referent	0.96 (0.79, 1.16)	0.95 (0.79, 1.15)
≥ 1/day	Referent	1.14 (0.87, 1.50)	1.05 (0.81, 1.37)
Legumes and pulses			
Never or <1/month	Referent	Referent	Referent
≥ 1/month but <1/week	Referent	0.92 (0.53, 1.58)	1.16 (0.66, 2.04)
≥ 1/week but <1/day	Referent	0.78 (0.52, 1.17)	0.69 (0.45, 1.06)
≥ 1/day	Referent	0.72 (0.48, 1.08)	0.87 (0.56, 1.34)
Fruit			
Never or <1/month	Referent	Referent	Referent
≥ 1/month but <1/week	Referent	1.14 (0.85, 1.53)	0.76 (0.55, 1.05)
≥ 1/week but <1/day	Referent	0.87 (0.67, 1.12)	0.72 (0.55, 0.95)
≥ 1/day	Referent	0.92 (0.69, 1.23)	1.03 (0.76, 1.38)
Vegetables^a^			
Never or <1/day	Referent	Referent	Referent
≥ 1/day	Referent	1.13 (0.97, 1.32)	1.28 (1.08, 1.50)
Whole grains			
Never or <1/month	Referent	Referent	Referent
≥ 1/month but <1/week	Referent	0.89 (0.68, 1.16)	0.93 (0.70, 1.23)
≥ 1/week but <1/day	Referent	0.80 (0.63, 1.02)	0.80 (0.62, 1.04)
≥ 1/day	Referent	0.93 (0.76, 1.14)	1.06 (0.86, 1.32)
Refined grains			
Never or <1/month	Referent	Referent	Referent
≥ 1/month but <1/week	Referent	1.18 (0.85, 1.64)	0.74 (0.52, 1.06)
≥ 1/week but <1/day	Referent	1.57 (1.22, 2.03)	1.40 (1.07, 1.82)
≥ 1/day	Referent	1.34 (1.03, 1.74)	1.56 (1.19, 2.04)
Desserts			
Never or <1/month	Referent	Referent	Referent
≥ 1/month but <1/week	Referent	1.13 (0.88, 1.45)	0.90 (0.70, 1.17)
≥ 1/week but <1/day	Referent	1.23 (0.98, 1.54)	0.91 (0.72, 1.15)
≥ 1/day	Referent	1.45 (1.10, 1.92)	1.15 (0.87, 1.52)
Deep-fried foods			
Never or <1/month	Referent	Referent	Referent
≥ 1/month but <1/week	Referent	1.09 (0.91, 1.31)	1.05 (0.87, 1.27)
≥ 1/week but <1/day	Referent	0.94 (0.79, 1.11)	0.90 (0.75, 1.08)
≥ 1/day	Referent	0.90 (0.66, 1.24)	0.84 (0.61, 1.17)
Fruit juice			
Never or <1/month	Referent	Referent	Referent
≥ 1/month but <1/week	Referent	0.88 (0.74, 1.06)	1.05 (0.87, 1.26)
≥ 1/week but <1/day	Referent	0.76 (0.64, 0.91)	0.92 (0.77, 1.09)
≥ 1/day	Referent	0.89 (0.68, 1.16)	1.01 (0.78, 1.31)
Sugar-sweetened beverages			
Never or <1/month	Referent	Referent	Referent
≥ 1/month but <1/week	Referent	0.85 (0.70, 1.02)	1.02 (0.83, 1.24)
≥ 1/week but <1/day	Referent	0.66 (0.56, 0.77)	0.97 (0.83, 1.14)
≥ 1/day	Referent	1.00 (0.71, 1.40)	1.23 (0.88, 1.73)

Values are odds ratios (95% confidence intervals) from multinomial logistic regression adjusted for age, household income, education, and tobacco and alcohol use

^a^Due to small sample sizes in the “Never or <1/month” and “≥1/month but <1/week” categories for vegetables, this dietary intake variable was modeled as a binary variable using logistic regression and the following two categories: “Never or <1/day” and “≥1/day”

In the unadjusted logistic regression model, participants in the highest tertile of full service and fast food restaurant density had significantly higher odds of overweight/obesity compared to participants in the lowest tertile: OR (95% CI), 1.44 (1.24, 1.67) (Table 3). After adjustment for age, household income, and education, the results were attenuated: OR (95% CI), 1.08 (0.92, 1.26). Further adjustment for tobacco and alcohol use, owning a vehicle, and moderate physical activity did not substantially impact effect estimates, nor did using an alternative South Asian cut-point for overweight/obese of BMI ≥23 kg/m^2^.

**Table 3:** Association between full service and fast food restaurant density (number of outlets per km^2^) and overweight/obesity among adults living in Delhi, India (*N* = 5264)

	Full service and fast food restaurant density (tertiles)		
	1 (*n* = 1737)	2 (*n* = 1782)	3 (*n* = 1745)
Overweight/obesity (BMI ≥25 kg/m^2^)			
Model 1: Unadjusted	Referent	1.03 (0.90, 1.20)	1.44 (1.24, 1.67)
Model 2: Adjusted for age, household income, and education	Referent	1.05 (0.90, 1.22)	1.08 (0.92, 1.26)
Model 3: Model 2 + tobacco and alcohol use	Referent	1.03 (0.88, 1.20)	1.09 (0.93, 1.27)
Model 4: Model 3 + moderate physical activity and owning a bicycle, car, or motorized bike	Referent	1.05 (0.90, 1.23)	1.07 (0.91, 1.25)
Overweight/obesity (BMI ≥23 kg/m^2^)			
Model 1: Unadjusted	Referent	1.09 (0.94, 1.27)	1.58 (1.35, 1.84)
Model 2: Adjusted for age, household income, and education	Referent	1.11 (0.95, 1.30)	1.18 (1.00, 1.39)
Model 3: Model 2 + tobacco and alcohol use	Referent	1.09 (0.93, 1.28)	1.18 (1.00, 1.40)
Model 4: Model 3 + moderate physical activity and owning a bicycle, car, or motorized bike	Referent	1.11 (0.95, 1.31)	1.16 (0.98, 1.38)

Values are odds ratios (95% confidence intervals) from logistic regression

When the outcome of BMI was modeled continuously, being in the highest versus lowest tertile of full service and fast food restaurant density was associated with a 1.1 kg/m^2^ increase in BMI (*p* < 0.0001). However, consistent with the models in which BMI was modeled as a binary outcome, this association was no longer significant after further adjustment for age, household income, and education: beta (SE), 0.2 (0.18), *p* = 0.18.

CEBs in the second and third tertile of wealth index were significantly (*p* < 0.0001) more likely to also have the highest tertile of full service and fast food restaurant density compared to CEBs in the first tertile of wealth index. The mean (SD) full service and fast food restaurant density for CEB wealth index tertile 1 was 2.50 (2.86) outlets per km^2^; for tertile 2 was 3.48 (3.09) outlets per km^2^; and for tertile 3 was 3.40 (2.08) outlets per km^2^. Within CEB wealth index tertiles, the mean BMI of participants did not increase with increasing CEB full service and fast food restaurant density tertile (Table 4).

**Table 4:** Mean (SD) body mass index (kg/m^2^) according to census enumeration block full service and fast food restaurant density and wealth index

		Census enumeration block wealth index		
		Tertile 1 (*n* = 1743)	Tertile 2 (*n* = 1792)	Tertile 3 (*n* = 1729)
Full service and fast food restaurant density	Tertile 1 (*n* = 1737)	23.20 (4.83) kg/m^2^	25.28 (5.14) kg/m^2^	27.63 (5.11) kg/m^2^
	Tertile 2 (*n* = 1704)	23.81 (4.87) kg/m^2^	25.27 (5.13) kg/m^2^	26.96 (5.18) kg/m^2^
	Tertile 3 (*n* = 1823)	23.15 (4.84) kg/m^2^	25.21 (4.97) kg/m^2^	27.84 (5.09) kg/m^2^

## Discussion

This is the first study to evaluate the relationship between the food environment, quantified as full service and fast food restaurant density, dietary intake, and overweight/obesity in India. After adjustment for age and socioeconomic factors, participants in the highest tertile of full service and fast food restaurant density were less likely to consume fruit and more likely to consume refined grains compared to participants in the lowest tertile. However, no significant association was observed between full service and fast food restaurant density and overweight/obesity or BMI. Participants living in areas with the highest full service and fast food restaurant density had higher household incomes and education, were more likely to have white collar employment, and more likely to own a car or motorized bike. These data therefore support that more affluent neighborhoods in urban megacities in developing countries are more likely to be undergoing the nutrition transition. More research is needed to improve our understanding of the food environment in urban areas of rapidly developing economies such as India.

This is among the first studies to report associations between the food environment and dietary intake in a LMIC. The previously mentioned study conducted in Brazil only evaluated the association of fast food restaurant density with sugar-sweetened beverage intake, and did not find a significant association [11]. Similarly, in our study, while in unadjusted models participants in the highest tertile of full service and fast food restaurant density were more likely to consume sugar-sweetened beverages compared to participants in the lowest tertile, this effect was attenuated after adjustment for confounders. A study of Japanese women found a significant association between intake of confectionaries and bread and availability of confectionaries and bread, but no associations with meat, fish, fruit and vegetables, or rice [28]. An inverse association was observed between fruit and vegetable intake and increasing density of fast food outlets among Australian children [29], which is similar to our observation of less frequent consumption of fruits among participants in the highest tertile of full service and fast food restaurant density in urban Indian adults. Together with the observation that these participants consumed refined grains more frequently, these results suggest that higher full service and fast food restaurant density may be associated with poor dietary intake in Delhi. To inform prevention interventions and programs, further research is needed, particularly in urban areas of LMICs, to clarify the relationship between access to restaurants and dietary intake.

A key finding of this study was the attenuation of the positive association between full service and fast food restaurant density and overweight/obesity after adjustment for socioeconomic factors such as household income and education. Several studies assessing food environment and BMI in LMICs such as Taiwan, Brazil, Japan, and South Korea also demonstrated no significant association after adjustment for socioeconomic status [11, 16, 18, 30]. We observed a strong, positive association between the wealth index of the neighborhood and full service and fast food restaurant density. This is in contrast to high-income countries such as the United States and Canada where areas with more fast food outlets tend to be the most deprived [31]. Longitudinal studies in larger, representative cohorts are needed to further disentangle the effects of the food environment on weight status in LMICs.

Many of the food outlets included in this dataset were Indian fast food, with few Western fast food outlets. A previous study in north India asked participants about their perceptions of fast food [32]. Chain restaurants were more likely to be considered fast food outlets by those from high-income neighborhoods [32]. 35% of individuals from high-income and 50% of individuals from low-income neighborhoods reported not eating at Western-style fast food restaurants [32]. The majority of individuals from both high- and low-income neighborhoods preferred home cooked food, and considered it to be healthier [32]. Our study adds to a growing literature that Westernization of the food system, often considered an indicator of the nutrition transition, may not be the only culprit for overweight/obesity in India. Thus, in addition to policies targeting large, multinational franchises, more research is needed to inform our understanding of how to address ready access to unhealthy food at Indian restaurants.

Previous research has focused on self-reported fast food consumption among urban Indian school children and risk of overweight/obesity [33–35]. One cross-sectional study conducted in the relatively small northern Indian city of Aligarh found that children (aged 10-16 years) who consumed fast food ≥1 time/week were more likely to be overweight/obese [33]. In contrast, a cross-sectional study in Delhi found that fast food consumption >3 times/week was negatively associated with BMI among adolescents [35]. Similarly, a cross-sectional study in the city of Chennai in southern India found that adolescents who consumed fast food 4-7 times/week were less likely to be overweight/obese compared to those who consumed fast food ≤3 times/week [34]. The authors hypothesized that the reason for these counterintuitive observations is reverse causality: overweight/obese adolescents are modifying their dietary behaviors in order to lose weight [34, 35].

There were several limitations of this study. We were unable to account for the temporal sequence because the GIS data were collected 2-3 years after the BMI data were obtained. In addition, street food is a common food outlet for people in urban India [36, 37], but no data on these facilities were collected in CARRS due to an inability to record the number of street food vendors that exist in an area since this varies hour-to-hour and day-to-day. Several other studies conducted in LMICs have also emphasized the need for data on street foods to appropriately capture how food environment relates to overweight/obesity because otherwise the food outlets included are mainly capturing the relationship for high-income areas [11, 16]. Furthermore, information on grocery stores and markets was not collected in our study. We also only evaluated the food environment surrounding participants’ residential address, but the food environment surrounding their workplace and a wider area surrounding their residence may influence participants’ food choices and weight status. This study may not be representative of other regions in India due to varying dietary patterns, built environment, and access to various foods across the country. Finally, we did not have information on fast food consumption or frequency of eating meals away from home. This information would provide further insight into the influence of the food environment on dietary intake.

## Conclusions

Most full service and fast food restaurants were Indian, suggesting that the nutrition transition in this megacity may be better characterized by the large number of unhealthy Indian food outlets rather than the Western food outlets. Full service and fast food restaurant density in the residence area of adults in Delhi, India, was associated with poor dietary intake. It was also positively associated with overweight/obesity, but this was largely explained by socioeconomic status. Prospective research is needed that explores these associations in other LMICs and across different regions in India, and that takes into account food outlets other than full service and fast food restaurants.

## Additional file

Additional file 1: Contains a figure of a heat map of body mass index levels overlaid on a map of the full service and fast food restaurants that was generated using ESRI ArcGIS software (Environmental Systems Research Institute, Redlands, CA). (JPEG 175 kb)

## Abbreviations

BMI: Body mass index
CARRS: Centre for Cardiometabolic Risk Reduction in South-Asia
CEB: Census enumeration block
CI: Confidence interval
CVD: Cardiovascular disease
GIS: Geographic information system
GPS: Global positioning system
LMICs: Low- and middle-income countries
OR: Odds ratio
