# Supplementary figures and images for: Association between full service and fast food restaurant density, dietary intake and overweight/obesity among adults in Delhi, India
Source: BMC Public Health. 2017 Jul 19;18:36. doi: 10.1186/s12889-017-4598-8 (PMC5518129; doi:10.1186/s12889-017-4598-8)

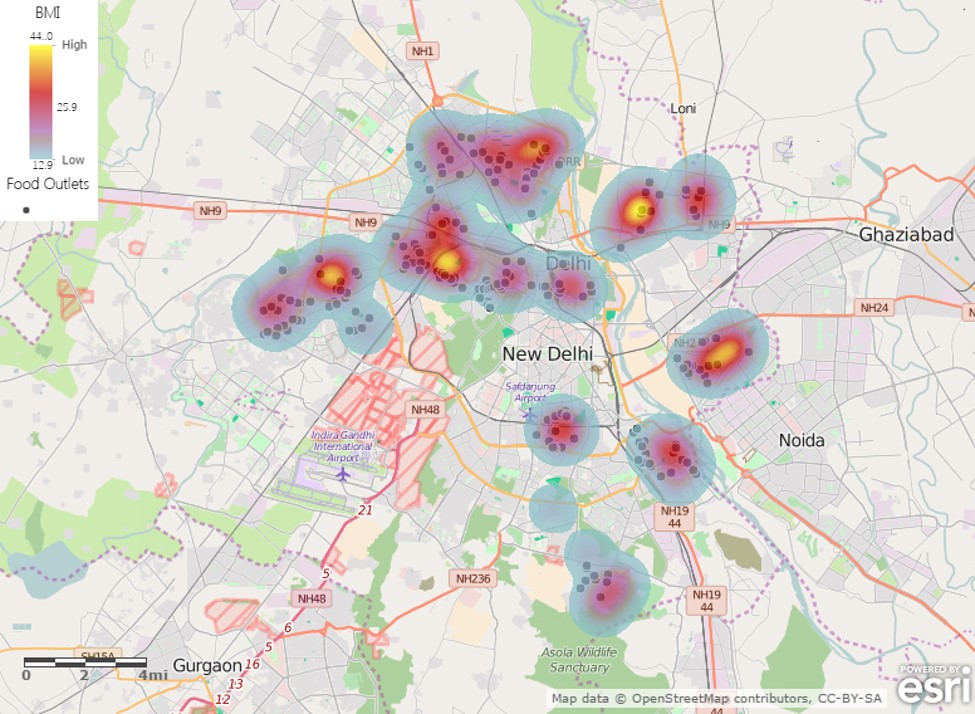

Supplement: Additional file 1: — Contains a figure of a heat map of body mass index levels overlaid on a map of the full service and fast food restaurants that was generated using ESRI ArcGIS software (Environmental Systems Research Institute, Redlands, CA). (JPEG 175 kb) [file 12889_2017_4598_MOESM1_ESM.jpg]
